# 2-Arylhydrazononitriles as building blocks in heterocyclic synthesis: A novel route to 2-substituted-1,2,3-triazoles and 1,2,3-triazolo[4,5-*b*]pyridines

**DOI:** 10.1186/1860-5397-3-12

**Published:** 2007-03-13

**Authors:** Saleh M Al-Mousawi, Moustafa Sh Moustafa

**Affiliations:** 1 Department of Chemistry, Faculty of Science; University of Kuwait: Safat; 13060: Kuwait, P.O. Box 12613

## Abstract

2-Arylhydrazono-3-oxobutanenitriles **2** was reacted with hydroxylamine hydrochloride to yield amidooxime **3**. This was cyclized into the corresponding oxadiazole **4** on refluxing in acetic anhydride. When refluxed in DMF in presence of piperidine, the corresponding 1,2,3-triazoleamine **5** was formed. The latter was acylated to **6** by addition of acetic anhydride while treatment of **5** with malononitrile gave the 1,2,3-triazolo [4,5-*b*]pyridine **8**. Treatment of acetyl derivative **6** with DMFDMA gave enaminone **9**. The enaminone **9** was coupled with benzenediazonium chloride to yield phenylazo-1,2,3-triazolo [4,5-*b*]pyridine **10**. Trials to convert compound **14** into 1,2,3-triazolo [4,5-*d*]pyrimidine **15** *via* refluxing in AcOH/NH_4_OAc failed. Instead the hydrolyzed product **5** was formed.

## Introduction

Interest in 1,2,3-triazole chemistry has recently been revived. [1–4] Zaprinast (**1**) is used as cyclic AMP phosphodiasterase inhibitor. [5] Plenty of 1,2,3-triazoles have also been reported to have useful pharmaceutical potential. [6–9] Synthetic approaches to 1,2,3-triazoloazines are rather limited. There are in fact few known routes to 4-functionally substituted 1,2,3-triazole-5-amines. [7] The latter is one of the logical starting points for condensed 1,2,3-triazoloazines. In the present article we provide a versatile route to these derivatives.

## Results and discussion

Elnagdi *et al* [10] have reported that arylhydrazononitriles reacted with hydroxylamine hydrochloride in ethanolic sodium acetate yielded amidooxime **3** that cyclized into aminoisoxazoles on treatment with mineral acids. Moreover, recently it was shown that refluxing **2a** with hydroxylamine hydrochloride in DMF/piperidine afforded 1,2,3-triazoles whose structure was established by X-ray crystal structure. [11] On the other hand reacting **2b** with hydroxylamine hydrochloride in DMF/piperidine afforded 5-amino-1,2,4-triazole *via* a rare Tiemann-like rearrangement. In order to clarify the rule of the substituent and the reaction condition we have investigated the behavior of compound **2c** with hydroxylamine hydrochloride in DMF/piperidine.

**Scheme 1:**
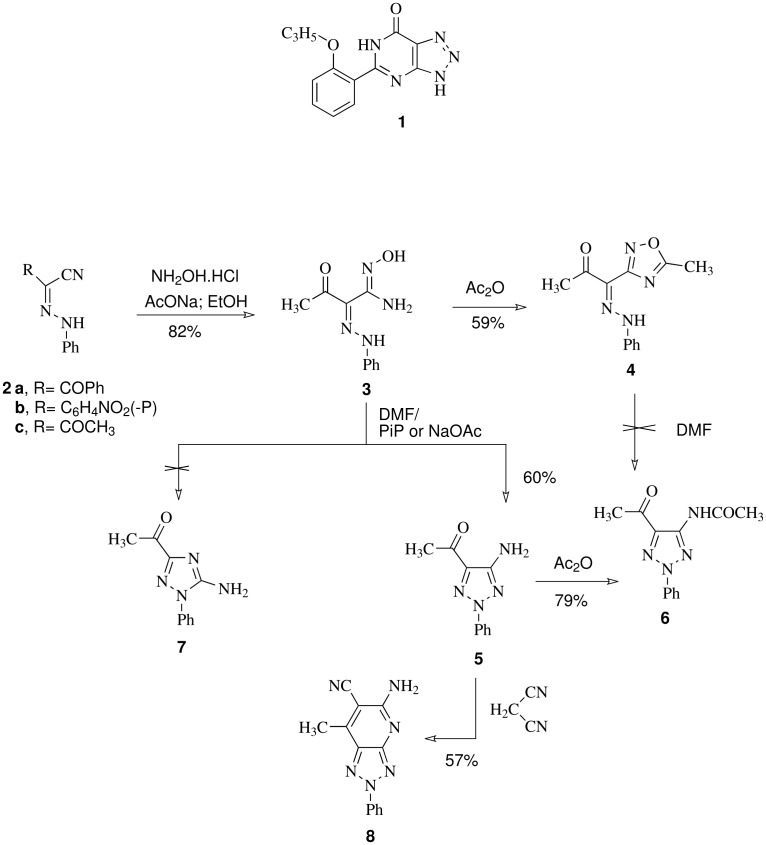
Synthesis of triazolopyridine **8**.

Reacting **2c** with hydroxylamine hydrochloride in ethanolic sodium acetate afforded the corresponding amidooxime **3** in good yield. Formation of amidooximes on reacting 2-arylhydrazononitriles with hydroxylamine hydrochloride under similar conditions has been reported earlier by Elnagdi *et al*. [10–11] Attempted cyclization of **3** into 1,2,3-triazole *via* reflux in acetic anhydride, as has been reported recently, [11] afforded a product that can be assigned as 1,2,4-oxadiazolylphenylhydrazone structure **4** or the isomeric acetylamino-1,2,3-triazole structure **6**. Phenylhydrazono-1,2,4-oxadiazoles have been reported to rearrange thermally into acylamino-1,2,3-triazoles. [12] Compound **5** could be obtained *via* refluxing **3** in DMF in the presence of piperidine or anhydrous sodium acetate. Acylation of the latter afforded the acetylamino-1,2,3-triazole **6** which proved to be different in all respects from **4**. To exclude the possible rearrangement of **3** prior to cyclization (Tiemann rearrangement), [13] as has been noted recently by Al-Matar *et al*, [14] and the possibility that the really formed product is the 5-amino-1,2,4-triazole **7.** We decided to investigate further the chemical reactivity pattern of the cyclized product (Scheme 1) to support our conclusion.

**Scheme 2:**
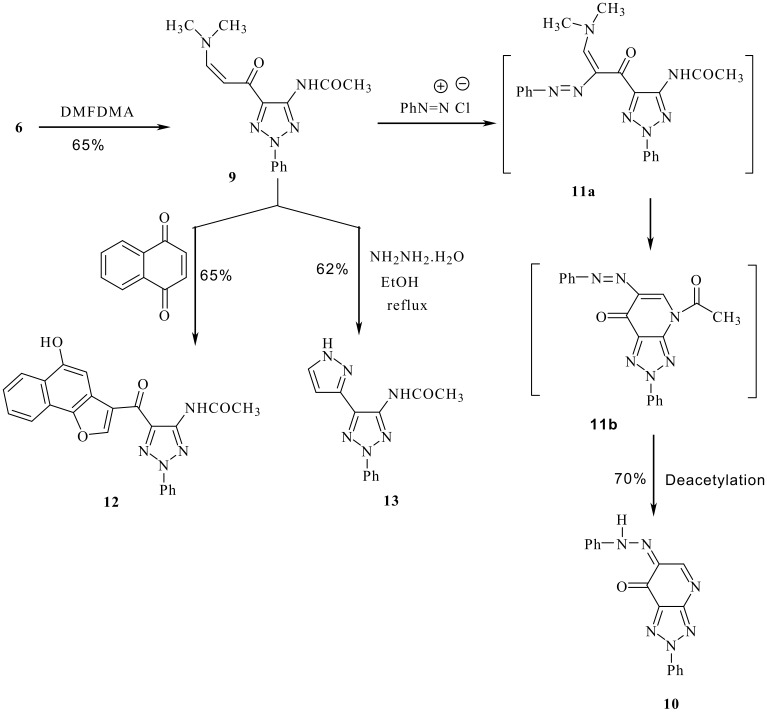
Synthesis of 1,2,3-triazoles and 1,2,3-triazolo[4,5-*b*]pyridines.

Compound **5** was reacted with malononitrile in refluxing DMF to yield the triazolopyridine **8**, thus confirming that the aminofunction and the acetyl function are adjacent. Moreover, reacting **6** with dimethylformamide dimethylacetal (DMFDMA) afforded the *cis* enaminone **9** (*J* = 8 Hz) despite the fact that secondary enaminones have been established to prefer adopting the *trans* configuration. [15] Compound **9** was coupled with benzenediazonium chloride most likely through the intermediate **11a**. The latter intermediate cyclized into **11b**. Deacetylation of **11b** followed by hydrogen shift produced **10**. This again confirms that the acetyl and the amino functions in cyclization product are adjacent as in **5** and therefore excluding **7** (Scheme 2). Typical of enaminones, compound **9** reacts with naphthoquinone to yield the benzofuran **12** and with hydrazine hydrate to yield pyrazolyl-1,2,3-triazole **13** in good yields.

**Scheme 3:**
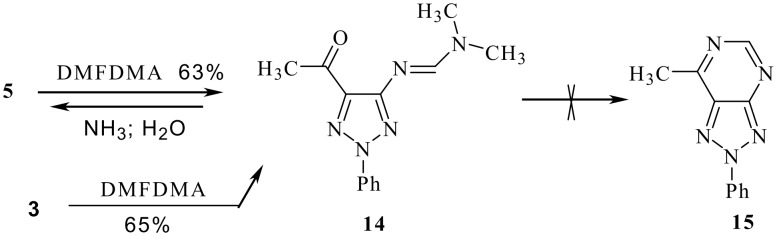
Identification of 1,2,3-triazole **14**.

Attempts to convert **4** into oxadiazolylhydrazone **6** by heating in DMF afforded **14** whose structure could be elucidated through synthesis independently from **3**. Trials to cyclise **14** into triazolopyrimidine **15** failed under a variety of conditions. It is thought that compound **15**, if formed, has hydrolyzed to **5** (Scheme 3).

## Conclusion

An excellent simple route to 2-substituted-1,2,3-triazols has been reached. It could be also revealed that the formed triazoles are excellent precursors to condensed triazoles and azolyltriazoles.

## Experimental Section

[See Supporting Information File 1]

## Supporting Information

File 1 Experimental Section. the experimental data and the results of analysis

## References

[1] Invidiata F P, Aiello S, Furno G, Aiello E, Simoni D, Rondanin R (2000). J Heterocycl Chem.

[2] Lalezari I, Gomez L A, Khorshidi M (1990). J Heterocycl Chem.

[3] Zhao Y B, Yan Z Y, Liang Y M (2006). Tetrahedron Lett.

[4] Kiselyou A S (2006). Tetrahedron Lett.

[5] Taniguchi Y, Tonai-Kuchi H, Shinjo K (2006). FEBS Lett.

[6] Kadaba P K (1988). J Med Chem.

[7] Wamhoff H, Potts K T (1984). Comprehensive Heterocyclic Chemistry.

[8] Ferrarini P L, Livi O (1981). Farmaco, Ed Sci.

[9] Matloubi H, Shafiee A, Saemian N, Shirvani G, Daha F J (2004). Appl Radiat Isot.

[10] Elnagdi M H, Elmoghayar M R H, Hafez E A A, Alnima H H (1975). J Org Chem.

[11] Ghozlan S A S, Abdelhamid I A, Elnagdi M H (2006). ARKIVOC.

[12] Fan W Q, Katritzky A R (1996). Comprehensive Heterocyclic Chemistry II.

[13] Tiemann F (1891). Ber Dtsch Chem Ges.

[14] Al-Matar H, Riyadh S M, Elnagdi M H (2006). Tetrahedron.

[15] Al-Saleh B, El-Apasery M A, Abdel-Aziz R S, Elnagdi M H (2005). J Heterocycl Chem.

