# Supplementary material for: 2-Arylhydrazononitriles as building blocks in heterocyclic synthesis: A novel route to 2-substituted-1,2,3-triazoles and 1,2,3-triazolo[4,5-b]pyridines
Source: Beilstein J Org Chem. 2007 Mar 13;3:12. doi: 10.1186/1860-5397-3-12 (PMC1847523; doi:10.1186/1860-5397-3-12)
Supplement: File 1 — Experimental Section. the experimental data and the results of analysis [file Beilstein_J_Org_Chem-03-12-s001.doc]

All melting points are uncorrected. Infrared spectra were recorded in KBr were determined on a Perkin-Elmer 2000 FT–IR system..^1^H NMR and ^13^C NMR spectra were determined on a Bruker DPX 400 MHz spectrometer in CDCl_3_ or DMSO-d_6_ as solvent and TMS as internal standard; chemical shifts are reported in *δ* (ppm). Mass spectra were measured on MS 30 and MS 9 (AEI) spectrometers, with EI 70 EV. Elemental analyses were measured by means of LEOCHNS-932 Elemental Analyzer.

**Synthesis of 3-oxo-2-(phenyl-hydrazono)-butyronitrile 2.**

A solution of 3-aminocrotononitrile (8.2 g, 0.1 mol) in ethanol (100 ml) was treated with sodium acetate (20 g). Benzene diazonium chloride was then added gradually, with stirring, to the mixture; the diazonium salt being prepared according to standard literature procedures from aniline (9.3 g, 0.1 mol), concentrated hydrochloric acid (2.5 ml), and sodium nitrite (6.9 g, 0.1 mol). After complete addition of the diazonium salt, the reaction mixture was kept at room temperature for one hour. The resulting solid product was filtered and dissolved in acetic acid (50 ml). The solution was then treated with concentrated hydrochloric acid (30 ml). The mixture was refluxed for 15 min, allowed to cool, and then poured onto ice-water. A yellow solid was obtained, which was isolated and recrystallized from ethanol to give yellow crystals; yield 86 %; mp. 177-178 ^0^C, ^1^H NMR 400 MHz (CDCl_3_) δ (ppm) = 2.5 (s, 3H, CH_3_), 7.2-7.4 (m, 5H, Ar-H), 9.6 (s, 1H, NH). ^13^C NMR (DMSO-d_6_) δ (ppm) = 193.5 (CO), 142.7, 130.3 (2C), 125.8, 117 (2C), 114.4, 111.8, 25.4 (CH_3_). MS: *m/z* (%) 187 (M^+^, 90), 144 (10), 92 (15), 77 (100); IR (KBr): υ_max_ = 3228 (NH), 2212 (CN), 1663 (CO); Anal.Calcd. for C_10_H_9_N_3_O (187.2): C, 64.1; H, 4.8; N, 22.4 %. Found C, 64.1; H, 4.8; N, 22.5 %.

**Synthesis of N-Hydroxy-3-oxo-2-(phenyl-hydrazono)-butyramidine 3.**

A mixture of **2** (1.87 g, 10 mmol), hydroxylamine hydrochloride (0.69 g; 10 mmol), and sodium acetate (0.82 g, 0.01mol) in ethanol (20 ml) was refluxed for 4h. After cooling the mixture was poured onto ice-water. The solid, so formed, was collected by filtration and crystallized from petroleum ether (60-80) to give yellow crystals; yield 82 %; mp. 185-186 ^0^C; ^1^H NMR 400 MHz (CDCl_3_): δ (ppm) = 2.5 (s, 3H, CH_3_), 6.1 (s,1H, OH) D_2_O exchanged, 6.5 (s, 2H, NH_2_) D_2_O exchanged, 7.1-7.4 (m, 5H, Ar-H), 13.4 (s, 1H, NH) D_2_O exchanged. ^13^C NMR (DMSO-d_6_): δ (ppm) = 199.5 (CO), 152.1, 143.3, 130.7 (2C), 127, 124.8, 115.9 (2C), 27.5 (CH_3_). MS: *m/z* (%) 220 (M^+^, 95), 203 (80), 187 (40), 160 (10), 117 (20), 77 (60); IR (KBr): υ_max_ = 3479 (OH), 3416, 3374 (NH_2_), 3286 (NH), 1685 (CO); Anal.Calcd. For C_10_H_12_N_4_O_2_ (220.23): C, 54.5; H, 5.5; N, 25.4 %. Found C, 54.8; H, 5.5; N, 25.4 %.

**Synthesis of 1-(5-methyl-[1,2,4]oxadiazol-3-yl)-1-(phenyl-hydrazono)-propan-2-one 4.**

Equimolecular amounts of **3** (2.2 g, 10 mmol) and acetic anhydride (1.02 g, 10 mmol) in acetic acid (20 ml) was refluxed for 2h. After cooling, the mixture was poured onto ice-water. The solid, so formed was collected by filtration and crystallized from petroleum ether (60-80) to give faint yellow crystals yield 59 %; mp. 115-117 ^0^C; ^1^H NMR 400 MHz (CDCl_3_): δ (ppm) = 2.3 (s, 3H, CH_3_), 2.7 (s, 3H, CH_3_), 7.2-7.5 (m, 3H, Ar-H), 8.1 (d, 2H, Ar-H), 10.2 (s, 1H, NH) D_2_O exchanged. ^13^C NMR (DMSO-d_6_): δ (ppm) = 192.7 (CO), 139.5, 130.9 (2C), 130.7, 129.7, 124.6, 119.7(2C), 19.2, 28.8 (CH_3_), 24.1 (CH_3_). MS: *m/z* (%) 244 (M^+^, 20), 202 (100), 186 (30), 160 (25), 118 (15), 77 (35); IR(KBr): υ_max_ = 3327 (NH), 1677 (CO); Anal.Calcd. for C_12_H_12_N_4_O_2_ (244.26): C, 59.0; H, 4.6; N, 23.0 %. Found C, 59.0; H, 4.9; N, 23.2 %.

**Synthesis of 1-(5-Amino-2-phenyl-2*H*-[1,2,3]triazol-4-yl)-ethanone 5.**

**Method A**: see ref.[11].

**Method B**: A mixture of compound **3** (2.2 g, 10 mmol), and N,N-dimethylformamide (10 ml) was treated with a few drops of piperidine. The reaction mixture was refluxed for 6-8 h., cooled and then poured onto ice-water. The solid, so formed was collected by filtration and crystallized from ethyl acetate to give yellow crystals; yield 60 %; mp. 125-127 ^0^C; ^1^H NMR 400 MHz (CDCl_3_): δ (ppm) = 2.6 (s, 3H, CH_3_), 5.0 (s,2H, NH_2_) D_2_O exchanged, 7.3 (t, 1H, Ar-H), 7.4 (t, 2H, Ar-H), 8 (d, 2H, Ar-H).^13^C NMR (DMSO-d_6_): δ (ppm) = 193.0 (CO), 155.3, 139.5, 132.7, 130.5 (2C), 128.6, 119.0 (2C), 12.0 (CH_3_). MS: m/z (%) 202 (M^+^, 100), 187 (25), 160 (20), 77 (30); IR(KBr): υ_max_ = 3409, 3324 (NH_2_); 1662 (CO); Anal.Calcd. For C_10_H_10_N_4_O (202.22) C, 59.4; H, 4.9; N,27.7 %. Found C, 59.4; H, 4.9; N, 27.4 %.

**Synthesis of N-(5-Acetyl-2-phenyl-2*H*-[1,2,3]triazol-4-yl)-acetamide 6.**

Equimolar amounts of compound **5** (2.02 g, 10 mmol) and acetic anhydride (1.02 g, 10 mmol) in acetic acid (20 ml) was refluxed for 2h. After cooling the reaction mixture was poured onto ice-water. The solid, so formed was collected by filtration and crystallized from petroleum ether (60-80) to give yellow crystals; yield 79 %; mp. 132-134 ^0^C; ^1^H NMR 400 MHz (DMSO-d_6_): δ (ppm) = 2.13 (s, 3H, CH_3_), 2.6 (s, 3H, CH_3_), 7.4-7.6 (m, 3H, Ar-H), 8 (d, 2H, Ar-H), 10.2 (s, 1H, NH) D_2_O exchanged. ^13^C NMR (CDCl_3_): δ (ppm) = 195 (CO), 181.2 (CO), 161.46, 147, 139, 130.1(2C), 129 (2C), 119.7, 26.7 (CH_3_), 25.1 (CH_3_). MS: *m/z* (%) 244 (M^+^, 40), 202 (100), 187 (20), 160 (25), 105 (10), 77 (25); IR (KBr): υ_max_ = 3328 (NH), 1720 (CO), 1670 (CO); Anal.Calcd. For C_12_H_12_N_4_O_2_ (244.25): C, 59.0; H, 4.9; N, 22.9 %. Found C, 59.1; H, 5.0; N, 22.7 %.

**Synthesis of 5-Amino-7-methyl-2-phenyl-2*H*-[1,2,3]triazolo[4,5-b] pyridine-6-carbonitrile 8.**

Equimolecular amounts of compound **5** (2.02 g, 10 mmol) and malononitrile (0.66 g, 10 mmol) in N,N-dimethylformamide (10 ml) were treatment with a few drops of piperidine. The reaction mixture was refluxed for 3h. After cooling the mixture was poured onto ice-water. The solid, so formed was collected by filtration and crystallized from petroleum ether (60-80) to give yellow crystals; yield 57%; mp. 230-232 ^0^C; ^1^H NMR 400 MHz (DMSO-d_6_): δ (ppm) = 2.8 (s, 3H, CH_3_), 6.6 (s, 2H, NH_2_) D_2_O exchanged, 7.2-8.3 (m, 5H, Ar-H). ^13^C NMR (DMSO-d_6_): δ (ppm) = 157, 153, 134, 131 (2C), 127, 125.5, 123, 120.8 (2C), 118.4, 82.4, 17.5 (CH_3_). MS: *m/z* (%) 250 (M^+^,100), 236 (65), 221 (10), 208 (10), 184 (10), 118 (10), 105 (10), 91 (25), 77 (45); IR (KBr): υ_max_ = 3329 (NH_2_), 2215 (CN); Anal.Calcd. For C_13_H_10_N_6_ (250.26): C, 62.4; H, 4.0; N, 33.5 %. Found C, 62.1; H, 4.2; N, 33.4 %.

**Synthesis of N-[5-(3-Dimethylamino-acryloyl)-2-phenyl-2H-[1,2,3]triazol-4-yl)-acetamide 9.**

A mixture of compound **6** (2.44 g, 10 mmol) and N,N-dimethylformamide dimethylacetal (1.19 g, 10 mmol) in xylene (20 ml) ) was refluxed for 6h. The reaction mixture was evaporated under reduced pressure in vacuum yielding a crude product, which was crystallized from toluene to give faint brown crystals; yield 65 %; mp.161-162 ^0^C; ^1^H NMR 400 MHz (CDCl_3_): δ (ppm) =2.3 (s, 3H, CH_3_), 3.0 (s, 3H, NCH_3_), 3.2 (s, 3H, NCH­_3_), 6.0 (d, 1H, j =8, CH), 7.3-7.5 (m, 3H, Ar-H), 7.8 (d, 1H, j =8, CH), 8.1 (d, 2H, Ar-H), 10.3 (s, 1H, NH) D_2_O exchanged; ^13^C NMR (CDCl_3_): δ (ppm) = 182.8 (CO), 167.7 (CO), 154.5, 147.2, 140, 134.9, 129.7 (2C), 128.3, 119.8(2C), 92.4, 46, 38.1, 25,1. IR (KBr): υ_max_ = 3278 (NH), 1688 (CO), 1636 (CO); MS: *m/z* (%) 299 (M^+^, 45), 282 (25), 256 (10), 240 (15), 212 (100), 187 (20), 152 (10), 77 (20); Anal.Calcd. for C_15_H_17_N_5_O_2_ (299.33): C, 60.1; H, 5.72; N, 23.4 %. Found C, 59.9; H, 5.6; N, 23.2%.

**Synthesis of 2-Phenyl-5-(phenyl-hydrazono)-2,5-dihydro-benzotriazol-4- one 10.**

A solution of compound **9** (2.99 g, 10 mmol) in ethanol (50 ml) was treated with sodium acetate (5 g). The aniline diazonium salt was added gradually with stirring to the mixture; The diazonium salt being prepared according to the stander literature procedures from aniline (0.93 g, 10 mmol), concentrated hydrochloric acid, and sodium nitrite. After complete addition of the diazonium salt, the reaction mixture was kept at room temperature for one hour. The solid, so formed was collected by filtration and crystallized from ethyl acetate to give dark yellow crystals; yield 70 %; mp. 263-265 ^0^C; ^1^H NMR 400 MHz (CDCl_3_): δ (ppm) = 7.4-7.6 (m, 8H, Ar-H), 8.3 (d, 2H, Ar-H), 8.6 (s, 1H, CH), 16.7 (s, 1H, NH) D_2_O exchanged; ^13^C NMR (CDCl_3_): δ (ppm) = 171.5 (CO), 161.2, 159.4, 141.4, 140.2, 135.8, 132.9, 130.6 (2C), 130 (2C), 130, 129.7, 120.8 (2C), 119.08 (2C). MS: *m/z* (%) 316 (M^+^, 100), 227 (10), 211 (20), 128 (35), 77 (85); IR (KBr): υ_max_ = 3435 (NH), 1626 (CO); Anal.Calcd. For C_17_H_12_N_6_O (316.32): C, 64.6 H, 3.8; N, 26.5 %. Found C, 64.7; H, 3.9; N, 26.3 %.

**Synthesis of N-[5-(5-Hydroxy-naphtho[1,2-b]furan-3-carbonyl)-2-phenyl-2*H*-[1,2,3]-triazol-4-yl]-acetamide 12.**

A mixture of compound **9** (2.99g, 10 mmol) and naphthoquinone (1.58g, 10 mmol) in glacial acetic acid (10 ml) was refluxed for 6h. After cooling the reaction mixture was poured onto ice-water. The solid, so formed was collected by filtration and crystallized from ethanol to give dark yellow crystals; yield 65 %; mp. 262-264 ^0^C; ^1^H NMR 400 MHz (DMSO-d_6_): δ (ppm) = 2.0 (s, 3H, CH_3_), 7.1-8.2(m, 10H, Ar-H,), 9.2 (s, 1H, Furyl-H), 10.3 (s,1H, OH) D_2_O exchanged,12.5 (s, 1H, NH) D_2_O exchanged;  ^13^C NMR (DMSO-d_6_): δ (ppm) = 181 (CO), 174 (CO), 155.2, 152.1, 150.1, 145.9, 145.3, 130.9 (2C), 130.6 (2C), 129.6, 126.1, 124.3, 122.3, 121.8 (2C), 121.7, 121.3 (2C), 111.6, 101.2, 24.1 (CH_3_). MS: *m/z* (%) 412 (M^+^, 40), 369 (80), 352 (60), 337 (20), 212 (70), 187 (20), 160 (20), 105 (20), 91 (40), 77 (95) ; IR (KBr): υ_max_ = 3442 (OH), 3234 (NH), 1679 (CO), 1630 (CO); Anal.Calcd. For C_23_H_16_N_4_O_4_ (412.4): C, 66.9; H, 3.9; N, 13.6 %. Found C, 67.1; H, 3.9; N, 13.8%.

**Synthesis of N-[2-phenyl-5-(1*H*-pyrazol-3-yl)-2*H*-[1,2,3]triazol-4-yl]-acetamide 13.**

Equimolecular amount of **9** (2.99 g, 10 mmol) and hydrazine hydrate (0.5 g, 10 mmol) in ethanol (20 ml) was refluxed for 8h. After cooling the reaction mixture was poured onto ice-water. The solid, so formed was collected by filtration and crystallized from ethanol to give yellow crystals; yield 62 %; mp. 236-237 ^0^C; ^1^H NMR 400 MHz (DMSO-d_6_): δ (ppm) = 2.1 (s, 3H, CH_3_), 6.6 (d, 1H, j =8 CH), 7.4-7.6 (m, 3H, Ar-H) 7.8 (d, 1H, j =8 CH), 8.0 (d, 2H, Ar-H), 10 (s, 1H, NH) D_2_O exchanged, 13.2 (s, 1H, NH) D_2_O exchanged; ^13^C NMR (DMSO-d_6_): δ (ppm) = 170 (CO), 142.2, 140, 138, 130.8 (2C), 128.5, 118.8 (2C), 117.66, 105.84, 104.52, 24.,8 (CH_3_). MS: *m/z* (%) 268 (M^+^, 80), 225 (95), 91 (40), 77 (45); IR (KBr): υ_max_ = 3443 (NH), 3319 (NH), 1697 (CO); ); Anal.Calcd. For C_13_H_12_N_6_O (268.28): C, 58.2; H, 4.5; N, 31.3 %. Found C, 58.5; H, 4.6; N, 31.1%.

**Synthesis of N\-[5-Acetyl-2-phenyl-2*H*-[1,2,3]triazol-4-yl)-N,N-dimethyl-formamidine 14.**

**Method A:** A mixture of compound **5** (2.02 g, 10 mmol) and N,N-dimethylformamide dimethylacetal (1.19 g, 10 mmol) in xylene (20 ml) was refluxed for 4-6h. The reaction mixture was evaporated under reduced pressure in vacuum yielding a crude product, which was crystallized from petroleum ether (60-80) to give yellow crystals; yield 63 %.

**Method B:** A mixture of compound **3** (2.2g, 10 mmol) and N,N-dimethylformamide- dimethylacetal (1.19g, 10 mmol) in xylene (20ml) was refluxed for 4-6h. The reaction mixture is evaporated under reduced pressure in vacuum yielded the crude product, which was crystallized from petroleum ether (60-80) to give yellow crystals m.p.130-131^0^C; yield 65%;

^1^H NMR 400 MHz (CDCl_3_): δ (ppm) = 2.6 (s, 3H, CH_3_), 3.1 (s, 3H, CH_3_), 3.2 (s, 3H, CH_3_), 7.3-7.5 (m, 3H, Ar-H), 8.1 (d, 2H, Ar-H), 8.4 (s, 1H, CH). ^13^C NMR (DMSO-d_6_): δ (ppm) = 193.0 (CO), 158.9, 157.8, 138.3, 129.8, 129.3 (2C), 128.3, 123.59 (2C), 41.4 (CH_3_), 35.4 (CH_3_), 27.0 (CH_3_). MS: *m/z* (%) 257 (M^+^, 100), 214 (50), 171 (20), 105 (15), 77 (45) ; IR (KBr): υ_max_ = 1679 (CO); Anal.Calcd. For C_13_H_15_N_5_O (257.3): C, 60.7; H, 5.9; N, 27.2 %. Found C, 60.7; H, 5.9; N, 27.0 %.
